# The snail *Biomphalaria glabrata* as a model to interrogate the molecular basis of complex human diseases

**DOI:** 10.1371/journal.pntd.0006552

**Published:** 2018-08-09

**Authors:** Joanna M. Bridger, Paul J. Brindley, Matty Knight

**Affiliations:** 1 Institute of Environment, Health, and Societies, Brunel University London, Uxbridge, United Kingdom; 2 Department of Microbiology, Immunology, and Tropical Medicine, School of Medicine and Health Sciences, George Washington University, Washington DC, United States of America; 3 Research Center for Neglected Diseases of Poverty, School of Medicine and Health Sciences, George Washington University, Washington DC, United States of America; 4 Division of Science and Mathematics, University of the District of Columbia, Washington DC, United States of America; PUCRS, BRAZIL

## Introduction

Schistosomiasis is considered the most important of the helminth diseases of humanity in terms of morbidity and mortality [1]. Although advances have been made in controlling the disease, long-term reduction remains elusive [2–5]. Schistosomiasis has re-emerged in southern Europe [6] where it had not been seen in recent times, unlike in more tropical endemic countries, including sub-Saharan Africa, the Maghreb, Egypt, and Brazil (http://www.thiswormyworld.org/worms/global-burden). These recent cases of schistosomiasis in higher latitudes suggest that global warming could influence the geographical range and snail susceptibility to infection as climate temperature increases.

The freshwater snail *Biomphalaria glabrata* has been studied for several years at the molecular level, mainly within the context of its interaction with the trematode *Schistosoma mansoni* for which it serves as the obligate intermediate host for asexual development of larval stages of the parasite. The genome sequence of *B*. *glabrata* has been reported [7], which reveals deep insights into the compatibility of this snail as a host for parasitism by *S*. *mansoni*. Analysis of the snail can be expected to further illuminate more deeply those molecular determinants of the snail that should give us insight into molecular interactions underlying the evolutionary success of this ancient relationship between the snail and schistosomes. Studies of comparative immunology relating to innate immunity have helped identify elements of invertebrate immunity that might also shape innate immunity in mammals. Hannington and colleagues recently reviewed this topic [8]. Here, we focus on aspects of the snail/schistosome relationship that could elucidate common pathways that enable both snails and humans to accommodate parasitism by schistosomes in the face of physiological and immunological environments.

## Studies of the snail: Schistosome interaction and spatial epigenetics in human infectious disease and cancer

Schistosomes induce stress in susceptible snails during early infection [14]. This difference was monitored closely over 24 hours following infection by the miracidium of *B*. *glabrata*, which revealed that intact but not radiation-attenuated miracidia induce stress in the susceptible snails [9]. The excretory/secretory products (ESPs) of the miracidium include the stress-inducing factor. With released ESPs, studies performed using an in vitro coculture system that utilized the *B*. *glabrata* embryonic (*Bg*e) cell line provided an opportunity to determine the effect of ESPs on interphase nuclei and nonrandom relocalization of gene loci and up-regulation of transcription after schistosome infection [15]. Subsequent investigation revealed that within a few minutes of invading the snail through the head-foot, schistosomes are capable of orchestrating, systemically, the nonrandom repositioning of specific gene loci in interphase nuclei of cells of the ovotestis (located in the posterior region of the snail), correlated positively with up-regulation of those specific genes [10]. Together, the phenomena observed with viable and attenuated schistosomes in snails that are either susceptible or resistant to infection revealed that the parasite coordinates the reorganization of the nuclear genome of its host, presumably to facilitate productive parasitism. Pathogenic bacteria and viruses can alter the epigenetic code of the host genome [11]. Epstein–Barr virus induces the repositioning of an entire chromosome in human B cells [12]. The consequences of altering gene location within nuclei include affecting its association with factors that regulate either gene expression or silencing [13]. Reorganization by a pathogen of the host genome could change the status of the gene expression profile in ways that facilitate infection [14].

**Fig 1:**
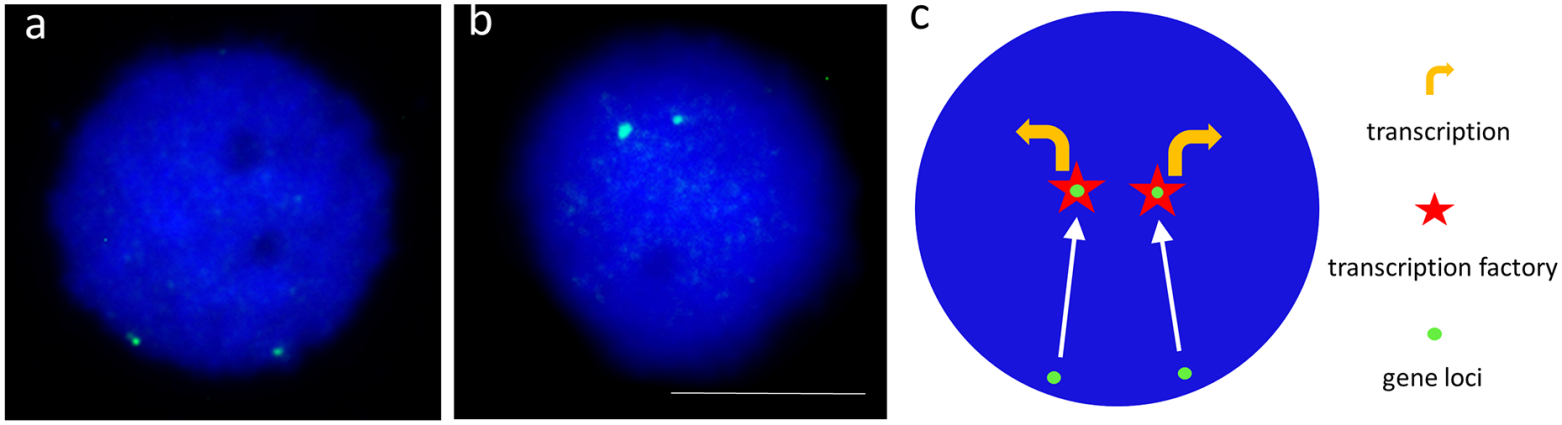
The movement of specific gene loci in *Biomphalaria glabrata* cell nuclei upon an exposure to miracidia of *Schistosoma mansoni*. Panels **a** and **b** display nuclei that have been extracted from tissues of snails before (a) and after (b) an exposure to schistosome miracidia (c). The blue fluorescent dye DAPI is used to delineate the nuclei as it intercalates into DNA and the Hsp70 gene loci (green), visualized with a fluorescent dye, after nuclei have been fixed and subjected to FISH, employing specific complimentary probes that bind exclusively to the gene of interest. Panel **d** is a cartoon representing the movement of gene loci seen after the stimulus of the infection. The white arrows represent the directed and active movement of the gene loci to transcription factories (red stars), with consequent up-regulation (yellow arrows). Scale bar 5 μm. FISH, fluorescence in situ hybridization; Hsp, heat shock protein.

How does this mechanism of pathogen-controlled genome reorganization facilitate productive parasitism by schistosomes in the snail *B*. *glabrata*? We speculate that signals from ESPs pass through cells from ESPs and are then communicated through the cytoskeletal network through the nuclear envelope, possibly via the linker of nucleoskeleton and cytoskeleton (LINC) complex. Once the signals reach the nucleus, epigenetic changes to the chromatin follow, in turn signaling specific gene loci to relocate to regions where transcription is up-regulated, for example, at a transcription factory [13]. Thus, the hypothesis we are following is that the parasite requires the gene products it has induced to become expressed for its own gain to elicit an infection. Our studies also revealed that the movement of the gene loci to new locations within the snail nuclei preceded the up-regulation of transcription of that particular gene [10]. Given that the gene encoding heat shock protein (Hsp) 70 moves early during infection [10, 15], we developed models that employ heat shock in both *Bg*e cells and intact snails to recapitulate the repositioning of the *Bg*Hsp70 gene. We have found that *Bg*Hsp70 gene loci relocate to new nonrandom locations within 1 hour, followed by Hsp70 expression. These *Bg*Hsp70 gene loci move into transcription factories, delineated by accumulations of RNA polymerase II staining [14, 16]. We presume that this facilitates transcription; although, this is yet to be established by delineating RNA in situ. Gene and chromosome repositioning can be blocked with agents that block nuclear myosin polymerization and by RNA interference targeting function of nuclear motor proteins within interphase nuclei [17, 18]. Interference prevents the specific gene relocation with consequential rapid down-regulation of transcription of the *Bg*Hsp70 gene in snails stressed by heat shock (Fig 1).

Gene repositioning in *B*. *glabrata* by schistosomes represents the first reported example of the influence of a eukaryotic pathogen in hijacking the genome behavior of its host. Since relocation and reprograming of gene loci is known in other human diseases, particularly during malignancy, signaling pathways involved in this chromosomal spatial epigenetics might be conveniently studied either in the snail/schistosome interaction or our snail/heat shock model of stress response. Other reports revealed specific nonrandom gene repositioning in cancer [13, 19]. These new locations of specific genes are so similar between individuals and cells that the new patterns of gene position within interphase nuclei can be utilized for diagnosis and prognosis in breast and prostate cancer [20]. These relocalized cancer genes include members of the heat shock family of stress proteins as well as other genes not normally involved in immunological responses.

**Fig 2:**
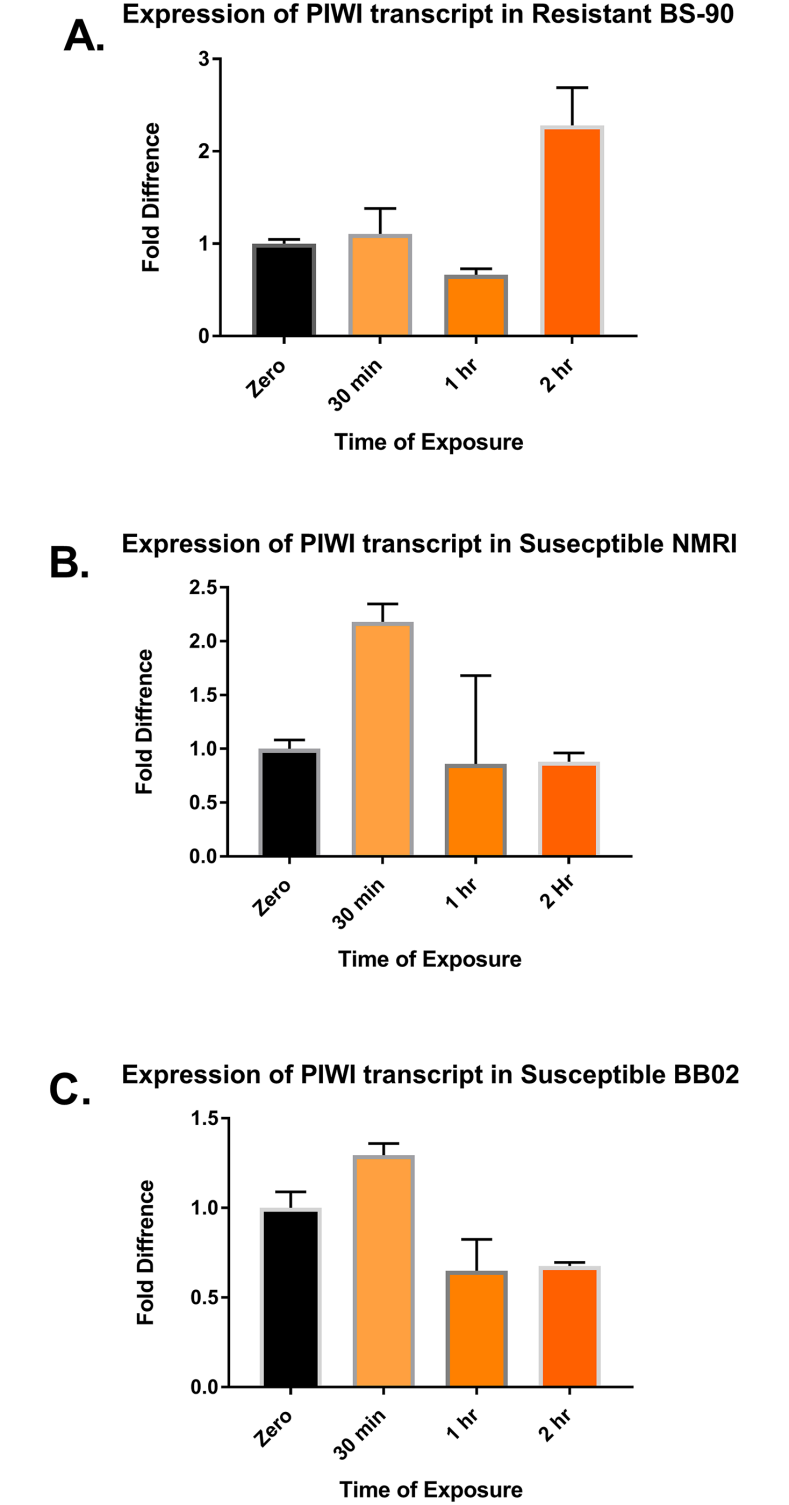
qPCR analysis shows that *piwi* RNA is differentially regulated in *S*. *mansoni* infected in *B*. *glabrata* snails depending on their susceptibility phenotypes as follows: Panel A, resistant BS-90 stock; B, susceptible NMRI stock; and C, susceptible BB02 stock. qPCR was performed as previously reported by Ittiprasert and colleagues (2009) with the following primers: 5′-GTCACACCTACCAGCTACAATG and 3′-GGTTCCCTGCCAGTTGAAATA. NMRI, Naval Medical Research Institute.

In preliminary studies, the analysis of expression of genes following infection of the snails with schistosomes revealed up-regulation of orthologues of cancer-related genes as an early (<60 min) response and could potentially be involved in chromatin reorganization. These included *c-myc*, which may participate in global genome reorganization [21, 22] and methyl transferases directly involved in chromatin remodeling. By contrast, both the genes *snail1* and *piwi* were down-regulated in the schistosome-susceptible snails and up-regulated in resistant snails (Fig 2). Both encode proteins involved in maintenance of heterochromatin [23, 24]. Indeed, *piwi* interacts directly with HP1a [23]. If *piwi* and HP1a are down-regulated in susceptible snails, genomic regions in nuclei of the snail could become more plastic, releasing many transcripts for expression. Spontaneous infective solid tumors have been found in mollusks, such as clams and mussels [25, 26], and up-regulation of p53 and *ras* transcription was detected in the cockle *Cerastoderma edule* with neoplasia [27]. With escalating interest in genome reorganization, including malignancy, we posit that mapping molecular pathways leading to spatial epigenetics in the snail–schistosome model represents an organism worthy of consideration to elucidate interplays between restructure of the nuclear architecture and pathogenesis of disease, given that it is facile, informative, inexpensive, and of minimal ethical concern.

## A model system for immunobiology in immunosuppression/immunostimulation involving stress proteins and lectins

**Fig 3:**
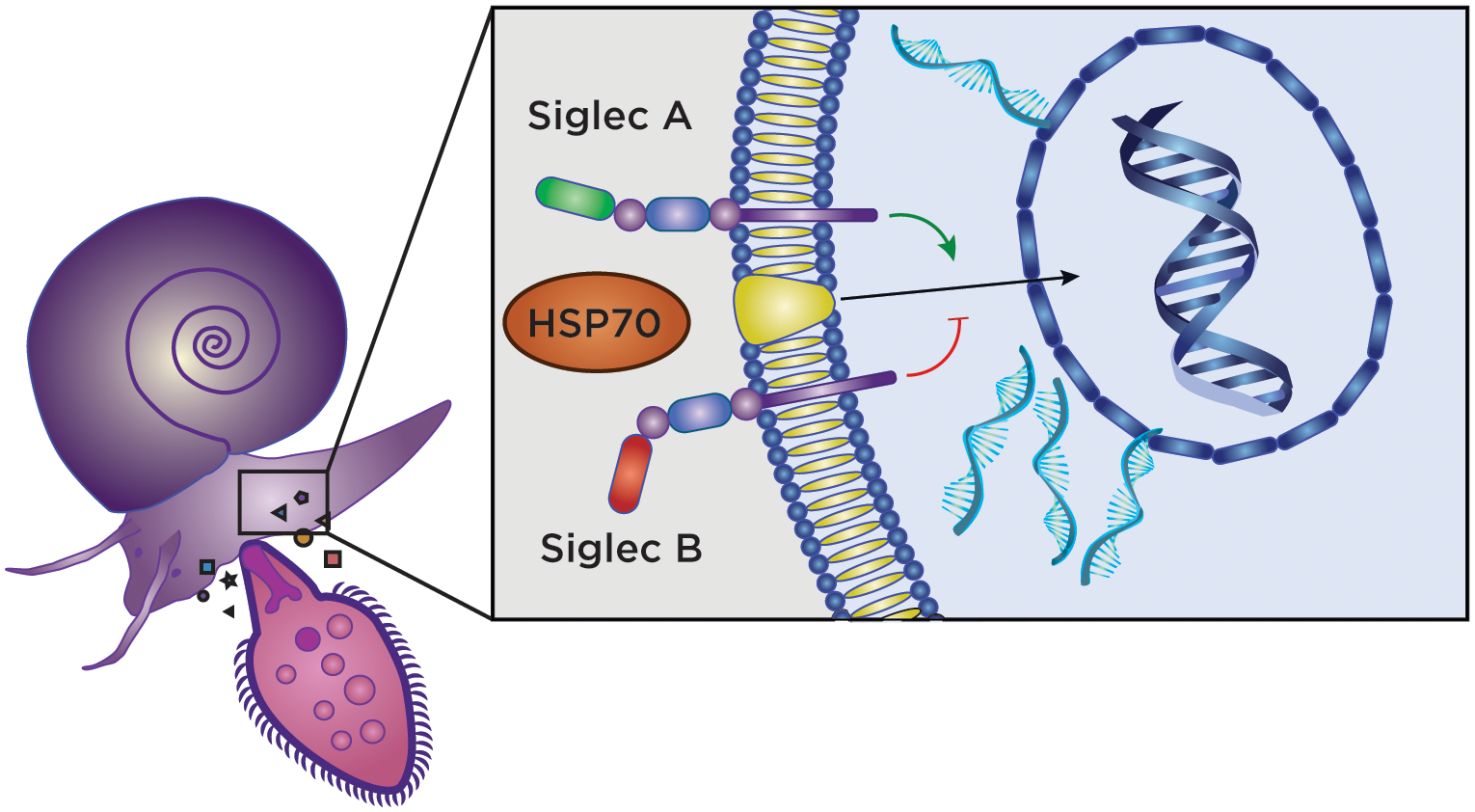
Working model with siglec-like–mediated signal transduction in *Biomphalaria glabrata* cells following stimulation by metabolites (hormone/peptides?) secreted by the schistosome miracidium during infection. We hypothesize that snail Hsp70 communicates with snail cell surface siglecs to mediate suppression of innate immune responses that, in turn, facilitates productive infection in susceptible genotypes of the snail. By contrast, siglec paralogues in resistant genotypes of the snail fail to transmit Hsp70 amplified signaling, and productive parasitism fails to be supported. Hsp, heat shock protein; siglecs, sialic acid-binding immunoglobulin like lectins.

Mechanism(s) used by lectins to influence the compatibility between the snail and schistosome are of increasing research focus [28–30]. These studies have shown that lectins from the snail and schistosomes are fundamental in compatibility issues. Lectins containing variable immunoglobulin domains, such as FREPs, have uncovered a sophisticated system of innate immunity that hinges on somatic rearrangement of these variable regions, leading to the diversification of these molecules. Variations in the structure and function contributing to a robust immune system against schistosomes in the snail offers the possibility of deciphering targets used to communicate via snail lectins to block infection. Recently, binding of stress protein Hsp70 to human siglecs 5 and 14 to either activate or suppress the immune system was reported [31]. Sialic acid-binding immunoglobulin like lectins (siglecs) are cell surface proteins that recognize sialoglycans [32]. Ongoing investigation is underway to characterize the association of Hsp70 of *B*. *glabrata* and snail homologs of siglecs to understand the connection between lectins, stress, and innate immunity. These studies provide a model whereby snail cell networks among existential stress proteins and lectins communicating in response to schistosomes could facilitate a better understanding of the role of these molecules in innate immunity in human and mammalian hosts of trematodes at large. Fig 3 presents an outline of a working model, reflecting the known situation in siglec-mediated signal transduction in human cells where Hsps are principal participants, binding lectin paralogues to either activate or suppress innate immune responses.

To conclude, model organisms have historically been used to simplify studies of complex mechanisms in cell and molecular biology. Mollusks including octopuses and the snails *Aplysia californica*, *IIyannasa obsoleta*, and *Crepidula fornicate* serve as models in neurobiology and developmental biology [33]. The availability of a reference genome, increasing molecular resources for functional genomics, and the *Bg*e cell line establish *B*. *glabrata* as an informative model for investigation of complex pathways involved in nuclear/genome behavior associated with infectious diseases and cancer.
